# Prognostic role of baseline hemoglobin level for long-term mortality in newly diagnosed rheumatoid arthritis: a cohort study

**DOI:** 10.3389/fnut.2025.1707271

**Published:** 2025-11-25

**Authors:** I-Wen Chen, Hsiu-Lan Weng, Shu-Wei Liao, Yi-Chen Lai, Kuei-Fen Wang, Jheng-Yan Wu, Ming-Chung Lin, Kuo-Chuan Hung

**Affiliations:** 1 Department of Anesthesiology, Chi Mei Medical Center, Liouying, Tainan, Taiwan; 2 Department of Anesthesiology, E-Da Hospital, I-Shou University, Kaohsiung, Taiwan; 3 Department of Anesthesiology, Chi Mei Medical Center, Tainan, Taiwan; 4 Center of General Education, Chia Nan University of Pharmacy and Science, Tainan, Taiwan; 5 Department of Nutrition, Chi Mei Medical Center, Tainan, Taiwan

**Keywords:** hemoglobin, anemia, rheumatoid arthritis, nutrition, hazard ratio, mortality

## Abstract

**Background:**

Patients with rheumatoid arthritis (RA) face an increased mortality risk compared to the general population. While anemia commonly occurs in patients with RA and is associated with disease activity in established diseases, the prognostic value of hemoglobin levels at initial diagnosis remains unknown. This study investigated whether baseline hemoglobin levels predict long-term mortality in patients with newly diagnosed RA.

**Methods:**

This retrospective cohort study utilized the TriNetX Analytics Network Platform to identify adult patients with newly diagnosed RA between 2010–2023. Patients were stratified by hemoglobin levels measured within 3 months of diagnosis: low hemoglobin group (< 12 g/dl) vs. control group (≥12 g/dl). Propensity score matching balanced the baseline characteristics and comorbidities. The primary outcome was 3-year overall mortality, with secondary outcomes including major cardiovascular events, stroke, intensive care unit (ICU) admission, severe anemia progression, and pneumonia.

**Results:**

After matching, 42,267 patients were included in each group. Low hemoglobin was independently associated with significantly increased 3-year mortality [Hazard ratio (HR): 1.80, 95% confidence interval (CI): 1.69–1.91, *p* < 0.001], with elevated risk already apparent at 1-year follow-up (HR: 1.90, 95% CI: 1.72–2.10). A clear dose-response relationship emerged: patients with hemoglobin 10–12 g/dl had a 68% increased mortality risk (HR: 1.68), while those with hemoglobin < 10 g/dl faced doubled risk (HR: 2.03). Low hemoglobin levels were also associated with elevated risks for major cardiovascular events (HR: 1.47), stroke (HR: 1.25), severe anemia progression (HR: 2.82), ICU admission (HR: 1.58), and pneumonia (HR: 1.43). The association remained consistent across subgroups and in contemporary cohorts (2018–2023).

**Conclusion:**

Low hemoglobin levels at RA diagnosis independently predict 3-year mortality with clear dose-response relationships. These findings support the incorporation of hemoglobin assessment into initial RA evaluation algorithms to identify high-risk patients warranting intensified monitoring and aggressive therapeutic intervention.

## 1 Introduction

Rheumatoid arthritis (RA) is a chronic autoimmune disease affecting ~0.5%−1% of the global population, characterized by systemic inflammation that drives both joint destruction and extra-articular manifestations (1–4). Despite therapeutic advances, mortality rates in RA patients remain 1.5 to 2 times higher than those in the general population, with cardiovascular complications and the cumulative burden of chronic inflammation being major contributors (5–7). Although various biomarkers have been proposed to assess disease severity and predict outcomes, identifying readily available clinical markers that reflect the inflammatory burden and predict long-term prognosis remains a critical unmet need (8–10). Anemia, occurring in 30%−60% of RA patients, has traditionally been viewed as a surrogate marker for systemic inflammatory burden rather than an independent pathological entity (11–14). The underlying mechanism involves inflammatory cytokines, particularly interleukin-6, which induces hepcidin production, subsequently impairing iron metabolism and erythropoiesis (15, 16). Recent evidence from a cohort study has confirmed that anemia in established RA patients is indeed associated with increased mortality risk, with iron deficiency anemia doubling all-cause mortality and non-iron deficiency anemia specifically increasing cardiovascular mortality (17). A recent study has also highlighted that hematologic indices, including the hemoglobin-to-red cell distribution width (RDW) ratio, are associated with inflammatory status and treatment response in RA patients prior to anti-TNF therapy (18).These findings suggest that anemia may reflect the severity of inflammatory processes that ultimately drive adverse outcomes.

However, a critical knowledge gap remains regarding the prognostic value of hemoglobin levels at the earliest stages of disease. While the study by Dong et al. (17) established the association between anemia and mortality in patients with established RA, whether hemoglobin levels measured at the time of initial RA diagnosis can serve as an early prognostic indicator for future outcomes remains unknown. This distinction is critically important; hemoglobin levels at diagnosis, prior to the confounding effects of long-term therapy or accumulated comorbidities, may offer a purer reflection of the underlying inflammatory burden. Therefore, this study aimed to investigate whether hemoglobin levels at RA diagnosis can predict long-term mortality risk using a large, multi-institutional database. Furthermore, in comparison with an osteoarthritis (OA) cohort, we sought to disentangle the independent and synergistic effects of systemic inflammation and anemia on long-term mortality.

## 2 Methods

### 2.1 Data source

This retrospective cohort study utilized the TriNetX Analytics Network Platform, a large-scale cloud-based healthcare database that aggregates de-identified electronic health records from over 120 healthcare organizations across 19 countries, including academic medical centers, specialty physician practices, and community hospitals (19). The platform encompasses more than 120 million patients and provides comprehensive clinical variables, including detailed demographics, diagnoses coded in the International Classification of Diseases format, medical procedures, laboratory values with temporal data, vital signs, and medication records. The database undergoes regular quality assurance processes and employs standardized data normalization procedures to ensure consistency across different electronic health record systems. This database has been widely used in numerous peer-reviewed clinical studies across various medical disciplines (20–23), demonstrating its reliability and validity for large-scale epidemiologic research. The Institutional Review Board of Chi Mei Medical Center approved this study protocol (IRB number: 11403-E01) and granted a waiver for informed consent requirements, recognizing that the retrospective analysis of de-identified data posed a minimal risk to patient privacy.

### 2.2 Patient population

We identified adult patients aged ≥ 18 years with newly diagnosed RA between January 1, 2010, and December 31, 2023, from the TriNetX database. The diagnosis of RA was established using the International Classification of Diseases, Tenth Revision (ICD-10) codes M05 and M06. To enhance diagnostic specificity, we further required that patients had initiated any conventional synthetic, biologic, or targeted synthetic disease-modifying antirheumatic drug (e.g., methotrexate, hydroxychloroquine, sulfasalazine) within 1 year after diagnosis (24). The date of RA diagnosis served as the index date. Patients were stratified into two cohorts based on hemoglobin levels measured within 3 months before or after the index date: the low hemoglobin (LHB) group included patients with hemoglobin levels below 12 g/dl, while the control group comprised those with hemoglobin levels of 12 g/dl or greater. Because of the analytic structure of the TriNetX platform, sex-specific anemia thresholds could not be simultaneously applied within a mixed-sex cohort. Separate analyses for males and females would substantially reduce sample size and limit comparability. Moreover, as the prevalence of RA is considerably higher among females, using a uniform cutoff of 12 g/dl minimized analytic bias and maintained consistency across groups.

### 2.3 Exclusion criteria

To minimize confounding from major comorbidities known to cause anemia or increase mortality risk, patients with a history of neoplasms (ICD-10 codes C00-D49) or advanced chronic kidney disease (stages 4 and 5, including end-stage renal disease) were excluded, as these diseases independently affect hemoglobin levels. Additionally, to minimize confounding from acute blood loss, we excluded patients with gastrointestinal bleeding, gastric ulcer, duodenal ulcer, peptic ulcer, or gastrojejunal ulcer diagnosed within 1 year before the index date. Patients with a prior diagnosis of heart failure (I50), cerebral infarction (I63), or liver cirrhosis (K74) were also excluded, as these diseases could independently influence both hemoglobin levels and mortality risk. While these criteria targeted major secondary causes of anemia, patients with uncomplicated pre-existing iron deficiency anemia were not explicitly excluded, thus representing a real-world clinical population.

### 2.4 Propensity score matching

Propensity score matching was employed to balance the baseline characteristics between the groups and minimize selection bias. Baseline characteristics were extracted from the 3-year period preceding the index date to ensure the capture of pre-existing comorbidities. The matching algorithm incorporated demographic factors, including age and race, body mass index, and comorbidities, such as hypertension, ischemic heart disease, diabetes mellitus, thyroid disorders, sleep disorders, liver diseases, cerebrovascular diseases, and systemic connective tissue disorders. Laboratory parameters integrated into the matching process included hemoglobin A1c values, serum albumin concentrations as indicators of both nutritional and inflammatory status, and estimated glomerular filtration rate. The matching utilized a greedy nearest-neighbor algorithm without replacement, implementing a caliper width of 0.1 standard deviations of the logit of the propensity score to ensure close matches while maintaining adequate sample size for robust statistical analysis.

### 2.5 Outcomes

The primary outcome was overall mortality within 3 years of the index date. Secondary outcomes included the risk of major cardiovascular events (defined as cardiac death, heart failure, or acute myocardial infarction), stroke, intensive care unit admission, development of severe anemia (hemoglobin below 10 g/dl), and pneumonia. To explore temporal relationships between hemoglobin levels and outcomes, we additionally evaluated all outcomes at 1 year post-index date. A 3-month washout period following the index date was implemented for all outcomes to minimize detection bias arising from increased medical surveillance immediately after RA diagnosis and to ensure adequate temporal separation between exposure and outcome ascertainment.

### 2.6 Sensitivity and subgroup analysis

We conducted a sensitivity analysis restricted to patients diagnosed with RA between 2018 and 2023 to examine whether the associations would remain consistent in a contemporary cohort with potentially improved therapeutic approaches. To explore dose-response relationships, we performed stratified analyses comparing patients with hemoglobin levels below 10 g/dl vs. those with levels between 10 and 12 g/dl, with the control group serving as the reference. Subgroup analyses were conducted to investigate whether the association between hemoglobin levels and mortality varied across different patient populations, stratified by age (18–50 years vs. over 50 years), sex, and presence of comorbidities including diabetes mellitus, hypertension, chronic kidney disease, dyslipidemia, and cardiovascular disease.

### 2.7 Additional analysis

To disentangle the effects of systemic inflammation from anemia, we introduced a comparison cohort of patients with newly diagnosed OA between 2010 and 2023 who had no history of RA. We stratified both the RA and OA cohorts by hemoglobin level (low: < 12 g/dl vs. normal: ≥12 g/dl). Using OA patients with normal hemoglobin as the universal reference group, we quantified three distinct mortality risks: (1) the risk from anemia alone (by comparing OA patients with low Hb to the reference), (2) the risk from RA inflammation alone (by comparing RA patients with normal hemoglobin to the reference), and (3) the combined synergistic risk of both exposures (by comparing RA patients with low hemoglobin to the reference).

### 2.8 Statistical analyses

Statistical analyses were performed using integrated analytical tools within the TriNetX platform. Baseline characteristics were described using means with standard deviations for continuous variables and frequencies with percentages for categorical variables. Balance after matching was evaluated using standardized mean differences, with values below 0.1 indicating adequate balance between groups. Kaplan-Meier estimates were used to explore event timing and cumulative incidence, with group differences assessed using log-rank tests. Hazard ratios with 95% confidence intervals were derived from Cox proportional hazards models, with the proportional hazards assumption verified using Schoenfeld residuals. All analyses followed an intention-to-treat approach, maintaining patients in their originally assigned groups regardless of subsequent changes in hemoglobin status during follow-up. To enhance methodological transparency and reproducibility, we have provided a supplemental table detailing all International Classification of Diseases, Tenth Revision, Clinical Modification (ICD-10-CM) codes used for variable definitions, inclusion and exclusion criteria, and outcome ascertainment (Supplementary Table 1). Statistical significance was defined as a two-sided *p*-value < 0.05.

## 3 Result

### 3.1 Patient selection and baseline characteristics

**Figure 1:**
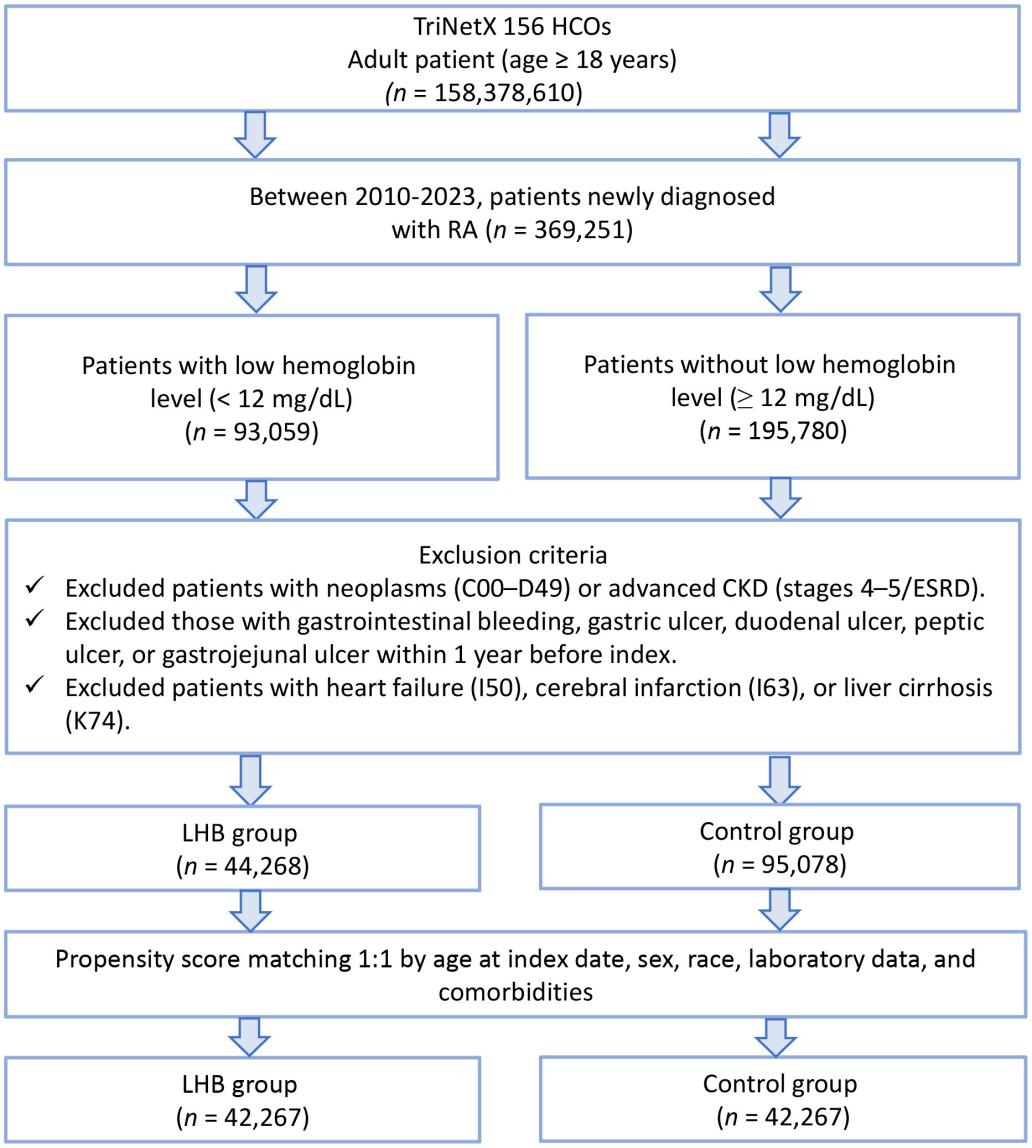
Patient selection flowchart from the TriNetX database. The flowchart illustrates the exclusion process applied to identify eligible patients with or without low hemoglobin (LHB) level.

From the TriNetX database, we identified 44,268 patients with newly diagnosed RA and low hemoglobin levels (< 12 g/dl) and 95,078 patients with normal hemoglobin levels (≥12 g/dl) between 2010 and 2023 (Figure 1). The initial cohorts showed substantial differences in baseline characteristics, with the low hemoglobin group being older (59.2 ± 17.7 vs. 54.4 ± 16.3 years) and having a higher proportion of females (80.1 vs. 69.9%; Table 1). After propensity score matching, we obtained a well-balanced cohort of 42,267 patients in each group. The matching successfully eliminated meaningful differences between the groups, with all standardized mean differences falling below 0.1, indicating excellent balance. The matched cohorts had nearly identical mean ages (58.8 ± 17.7 vs. 58.4 ± 16.7 years) and similar distributions of comorbidities including hypertension (28.1 vs. 27.8%), diabetes mellitus (11.4 vs. 10.8%), and ischemic heart disease (8.0 vs. 7.7%). Medication use was also comparable (Supplementary Table 2).

**Table 1:** Baseline characteristics of newly diagnosed rheumatoid arthritis patients by hemoglobin level before and after propensity score matching.

**Variables**	**Before matching**			**After matching**		
	**LHB group (*****n*** = **44,268)**	**Control group (*****n*** = **95,078)**	**SMD** ^†^	**LHB group (*****n*** = **42,267)**	**Control group (*****n*** = **42,267)**	**SMD** ^†^
**Patient characteristics**						
Age at index (years)	59.2 ± 17.7	54.4 ± 16.3	0.280	58.8 ± 17.7	58.4 ± 16.7	0.020
Female (%)	35,440 (80.1%)	66,436 (69.9%)	0.237	33,634 (79.6%)	34,193 (80.9%)	0.033
BMI≥30 kg/m^2^	10,259 (23.2%)	23,497 (24.7%)	0.036	9,767 (23.1%)	9,677 (22.9%)	0.005
White	25,224 (57.0%)	62,995 (66.3%)	0.192	24,424 (57.8%)	23,762 (56.2%)	0.032
Black or African American	7,627 (17.2%)	8,870 (9.3%)	0.234	6,788 (16.1%)	7,012 (16.6%)	0.014
Unknown race	6,875 (15.5%)	13,979 (14.7%)	0.023	6,680 (15.8%)	6,982 (16.5%)	0.019
Asian	2,409 (5.4%)	4,686 (4.9%)	0.023	2,330 (5.5%)	2,456 (5.8%)	0.013
other race	1,700 (3.8%)	3,479 (3.7%)	0.010	1,625 (3.8%)	1,649 (3.9%)	0.003
Factors influencing health status and contact with health services	23,022 (52.0%)	44,872 (47.2%)	0.096	21,510 (50.9%)	21,534 (50.9%)	0.001
**Comorbidities**						
Essential (primary) hypertension	13,233 (29.9%)	19,177 (20.2%)	0.226	11,886 (28.1%)	11,742 (27.8%)	0.008
Dyslipidemia	8,424 (19.0%)	14,614 (15.4%)	0.097	7,721 (18.3%)	7,489 (17.7%)	0.014
Systemic connective tissue disorders	6,447 (14.6%)	11,723 (12.3%)	0.066	6,043 (14.3%)	6,317 (14.9%)	0.018
Disorders of thyroid gland	5,402 (12.2%)	9,143 (9.6%)	0.083	4,946 (11.7%)	4,888 (11.6%)	0.004
Diabetes mellitus	5,503 (12.4%)	7,326 (7.7%)	0.158	4,812 (11.4%)	4,547 (10.8%)	0.020
Overweight and obesity	4,671 (10.6%)	7,687 (8.1%)	0.085	4,272 (10.1%)	4,199 (9.9%)	0.006
Sleep disorders	3,982 (9.0%)	7,948 (8.4%)	0.023	3,707 (8.8%)	3,683 (8.7%)	0.002
Ischemic heart diseases	4,046 (9.1%)	4,427 (4.7%)	0.178	3,395 (8.0%)	3,234 (7.7%)	0.014
Nicotine dependence	2,575 (5.8%)	5,052 (5.3%)	0.022	2,393 (5.7%)	2,423 (5.7%)	0.003
Other chronic obstructive pulmonary disease	2,474 (5.6%)	2,842 (3.0%)	0.129	2,119 (5.0%)	2,013 (4.8%)	0.012
Chronic kidney disease (CKD)	2,423 (5.5%)	2,117 (2.2%)	0.169	1,962 (4.6%)	1,746 (4.1%)	0.025
Cerebrovascular diseases	1,825 (4.1%)	1,735 (1.8%)	0.136	1,470 (3.5%)	1,374 (3.3%)	0.013
Diseases of liver	1,583 (3.6%)	2,564 (2.7%)	0.050	1,445 (3.4%)	1,486 (3.5%)	0.005
COVID-19	894 (2.0%)	1,627 (1.7%)	0.023	827 (2.0%)	828 (2.0%)	0.000
Malnutrition	945 (2.1%)	597 (0.6%)	0.129	706 (1.7%)	548 (1.3%)	0.031
Pulmonary fibrosis	696 (1.6%)	904 (1.0%)	0.056	616 (1.5%)	604 (1.4%)	0.002
Alcohol related disorders	502 (1.1%)	876 (0.9%)	0.021	458 (1.1%)	467 (1.1%)	0.002
Disorders of parathyroid gland	204 (0.5%)	320 (0.3%)	0.020	184 (0.4%)	182 (0.4%)	0.001
**Laboratory data**						
Hemoglobin A1c ≥7%	1,776 (4.0%)	2,908 (3.1%)	0.052	1,596 (3.8%)	1,581 (3.7%)	0.002
Albumin g/dl (≥3.5 g/dl)	18,064 (40.8%)	41,036 (43.2%)	0.048	17,221 (40.7%)	17,713 (41.9%)	0.024
eGFR >60 ml/min/1.73 m^2^	22,150 (50.0%)	45,421 (47.8%)	0.045	20,921 (49.5%)	21,455 (50.8%)	0.025

IDA, iron deficiency anemia; BMI, body mass index; SMD, standardized mean differences; eGFR, estimated glomerular filtration rate. ^†^SMD values < 0.1 indicate adequate balance between groups.

### 3.2 Association between low hemoglobin and 3-year outcomes

The primary analysis revealed a significant association between low hemoglobin levels and mortality risk (Table 2). Patients with low hemoglobin levels experienced significantly higher overall mortality at 3 years (HR: 1.80, 95% CI: 1.69–1.91, *p* < 0.001), highlighting the prognostic importance of this readily available laboratory marker. Beyond overall mortality, low hemoglobin levels were consistently associated with elevated risks across all secondary outcomes. Cardiocerebral complications were markedly higher in the LHB group than in the controls, including major cardiovascular events (HR: 1.47; 95% CI: 1.40–1.55; *p* < 0.001) and stroke (HR: 1.25; 95% CI: 1.14–1.37; *p* < 0.001). Of particular concern was the pronounced increase in severe anemia (hemoglobin < 10 g/dl) during follow-up; 24.4% of patients with initially low hemoglobin progressed to severe anemia compared with only 10.6% of controls (HR: 2.82; 95% CI: 2.73–2.92).

**Table 2:** Three-year clinical outcomes in newly diagnosed rheumatoid arthritis patients by baseline hemoglobin level.

**Outcomes**	**LHB group (*****n***= **42,267)**		**Control group (*****n***= **42,267)**		**HR (95% CI)**	***p*-Value**
	**Events (** ***n*** **)**	**Incidence (%)**	**Events (** ***n*** **)**	**Incidence (%)**		
Overall mortality	2,611	6.2	1,610	3.8	1.80 (1.69–1.91)	< 0.001
Major cardiovascular events	3,860	9.1	2,920	6.9	1.47 (1.40–1.55)	< 0.001
Stroke	976	2.3	861	2.0	1.25 (1.14–1.37)	< 0.001
ICU admission	1,912	4.5	1,344	3.2	1.58 (1.47–1.69)	< 0.001
Severe anemia	10,305	24.4	4,464	10.6	2.82 (2.73–2.92)	< 0.001
Pneumonia	3,034	7.2	2,367	5.6	1.43 (1.35–1.51)	< 0.001

LHB, low hemoglobin; ICU, intensive care unit; HR, hazard ratio; CI, confidence interval.

For the 1-year follow-up (Table 3), patients with low hemoglobin levels showed significantly higher 1-year mortality (HR: 1.90, 95% CI: 1.72–2.10, *p* < 0.001), with similar elevations in major cardiovascular events (HR: 1.57) and ICU admissions (HR: 1.66). Notably, the risk of severe anemia progression was even more pronounced at 1 year (HR: 3.40, 95% CI: 3.24–3.56), with 15.5% of patients with low hemoglobin progressing to severe anemia compared to 5.3% of controls.

**Table 3:** One-year clinical outcomes in newly diagnosed rheumatoid arthritis patients by baseline hemoglobin level.

**Outcomes**	**LHB group (*****n***= **42,267)**		**Control group (*****n***= **42,267)**		**HR (95% CI)**	***p*-Value**
	**Events (** ***n*** **)**	**Incidence (%)**	**Events (** ***n*** **)**	**Incidence (%)**		
Overall mortality	1,031	2.4	588	1.4	1.90 (1.72–2.10)	< 0.001
Major cardiovascular events	2,135	5.1	1,482	3.5	1.57 (1.47–1.67)	< 0.001
Stroke	512	1.2	440	1.04	1.25 (1.10–1.42)	< 0.001
ICU admission	891	2.1	581	1.4	1.66 (1.50–1.84)	< 0.001
Severe anemia	6,567	15.5	2,231	5.3	3.40 (3.24–3.56)	< 0.001
Pneumonia	1,471	3.5	1,039	2.5	1.54 (1.42–1.66)	< 0.001

LHB, low hemoglobin; ICU, intensive care unit; HR, hazard ratio; CI: confidence interval.

### 3.3 Sensitivity and dose-response analyses

To validate our findings in a contemporary cohort with potentially improved RA management strategies, we performed a sensitivity analysis restricted to patients diagnosed between 2018 and 2023. This analysis of 24,413 matched pairs demonstrated remarkably consistent results, with patients with low hemoglobin levels maintaining an elevated mortality risk (HR: 1.83, 95% CI: 1.67–2.00; Table 4). The persistence of this association in recent years suggests that despite advances in RA treatment, the prognostic significance of low hemoglobin levels remains relevant.

**Table 4:** Three-year clinical outcomes in contemporary cohort (2018–2023): sensitivity analysis.

**Outcomes**	**LHB group (*****n***= **24,413)**		**Control group (*****n***= **24,413)**		**HR (95% CI)**	***p*-Value**
	**Events (** ***n*** **)**	**Incidence (%)**	**Events (** ***n*** **)**	**Incidence (%)**		
Overall mortality	1,222	5.0	757	3.1	1.83 (1.67–2.00)	< 0.001
Major cardiovascular events	2,265	9.3	1,689	6.9	1.53 (1.44–1.63)	< 0.001
Stroke	544	2.2	480	1.97	1.27 (1.13–1.44)	< 0.001
ICU admission	1,097	4.5	780	3.2	1.60 (1.46–1.75)	< 0.001
Severe anemia	5,643	23.1	2,445	10.0	2.87 (2.74–3.01)	< 0.001
Pneumonia	1,630	6.7	1,328	5.4	1.40 (1.30–1.50)	< 0.001

LHB, low hemoglobin; ICU, intensive care unit; HR, hazard ratio; CI, confidence interval.

The dose-response analysis provided additional insights into the relationship between hemoglobin levels and outcomes (Table 5). When stratifying the patients into three groups, we observed a clear gradient of risk. Compared to patients with hemoglobin ≥12 g/dl, those with hemoglobin 10–12 g/dl had a 68% increased mortality risk (HR: 1.68, 95% CI: 1.57–1.79), while those with hemoglobin < 10 g/dl faced a doubled risk (HR: 2.03, 95% CI: 1.87–2.21). This dose-response relationship strengthens the causal inference and suggests that even mild reductions in hemoglobin carry clinical significance in patients with RA.

**Table 5:** Dose-response relationship between baseline hemoglobin categories and 3-year clinical outcomes.

**Outcomes**	**Hb 10–12 g/dl (*****n*** = **40,087 for each group)**		**Hb**<**10 g/dl (*****n*** =**16,822 for each group)**	
	**HR (95% CI)**	***p*** **-Values**	**HR (95% CI)**	***p*** **-Values**
Overall mortality	1.68 (1.57–1.79)	< 0.001	2.03 (1.87–2.21)	< 0.001
Major cardiovascular events	1.38 (1.31–1.45)	< 0.001	1.48 (1.39–1.59)	< 0.001
Stroke	1.19 (1.08–1.31)	< 0.001	1.15 (1.01–1.30)	0.034
ICU admission	1.40 (1.30–1.50)	< 0.001	1.68 (1.53–1.85)	< 0.001
Severe anemia	2.40 (2.31–2.49)	< 0.001	3.62 (3.44–3.80)	< 0.001
Pneumonia	1.35 (1.27–1.42)	< 0.001	1.46 (1.35–1.58)	< 0.001

Hb, hemoglobin; ICU, intensive care unit; HR, hazard ratio; CI, confidence interval.

### 3.4 Subgroup analyses

While the association remained significant across all examined subgroups, interesting patterns emerged (Table 6). Patients without diabetes showed a stronger association (HR: 1.75) than those with diabetes (HR: 1.33, *p* for interaction = 0.002). Similarly, patients without hypertension demonstrated a stronger association (HR, 1.78) than those with hypertension (HR: 1.53, *p* for interaction = 0.033). When stratified by age, patients aged 18–50 years (HR: 1.68) and those aged > 50 years (HR: 1.71) both exhibited significantly elevated mortality risks, with no significant interaction between the two age groups (*p* = 0.904).

**Table 6:** Subgroup analysis of association between low hemoglobin and 3-year mortality risk.

**Subgroup analysis (*n* for each group)**	**HR (95% CI)**	***p*-Value**	***p* for interaction**
**Sex**			
Male, (*n* = 7,519)	1.80 (1.60–2.02)	< 0.001	Reference
Female, (*n* = 33,808)	1.74 (1.61–1.87)	< 0.001	0.634
**Age**			
18–50 years (*n* = 8,656)	1.68 (1.27–2.22)	< 0.001	Reference
>50 years, (*n* = 33,177)	1.71 (1.61–1.82)	< 0.001	0.904
**Diabetes mellitus**			
Yes, (*n* = 3,622)	1.33 (1.11–1.60)	0.002	Reference
No, (*n* = 38,367)	1.75 (1.64–1.87)	< 0.001	0.002
**Hypertension**			
Yes, (*n* = 8,530)	1.53 (1.35–1.73)	< 0.001	Reference
No, (*n* = 32,783)	1.78 (1.66–1.92)	< 0.001	0.033
**Dyslipidemia**			
Yes, (*n* = 4,071)	1.55 (1.31–1.85)	< 0.001	Reference
No, (*n* = 37,828)	1.84 (1.72–1.97)	< 0.001	0.056
**Chronic kidney disease**			
Yes, (*n* = 1,248)	1.40 (1.06–1.85)	0.017	Reference
No, (*n* = 40,864)	1.77 (1.66–1.89)	< 0.001	0.078
**Cardiovascular disease**			
Yes, (*n* = 2,352)	1.56 (1.29–1.90)	< 0.001	reference
No, (*n* = 39,501)	1.81 (1.69–1.93)	< 0.001	0.135

HR, hazard ratio; CI, confidence interval.

### 3.5 Additional analysis

Using patients with OA and normal hemoglobin as a reference, we found that low hemoglobin alone increased the mortality risk by 49% (HR: 1.49), while RA with normal hemoglobin increased the risk by 40% (HR: 1.40; Table 7). However, the combination of RA and low hemoglobin levels resulted in a 130% increased mortality risk (HR: 2.30), suggesting a synergistic effect between inflammatory arthritis and anemia. This multiplicative rather than additive risk pattern indicates that low hemoglobin in RA patients represents more than simple anemia; it likely reflects the severity of the underlying inflammatory processes that drive both anemia and adverse outcomes.

**Table 7:** Comparative mortality risk analysis: rheumatoid arthritis vs. osteoarthritis by hemoglobin status.

**Cohort**	**HR (95% CI)**	***p*-Values**	***N* for each group**
OA with Hb ≥12 g/dl	1	–	–
OA with Hb < 12 g/dl	1.49 (1.47–1.52)	< 0.001	703,046
RA with Hb ≥12 g/dl	1.40 (1.32–1.48)	< 0.001	95,074
RA with Hb < 12 g/dl	2.30 (2.16–2.46)	< 0.001	44,246

OA, osteoarthritis; RA, rheumatic arthritis; HR, hazard ratio; CI, confidence interval.

## 4 Discussion

This large cohort study of 42,267 matched pairs demonstrated that low hemoglobin levels at RA diagnosis are independently associated with an 80% increased risk of 3-year mortality. The association exhibited a clear dose-response relationship, with the mortality risk escalating from 68% for mild anemia to double the risk for severe anemia. Notably, the prognostic impact of low hemoglobin levels was particularly pronounced in patients without major comorbidities and remained consistent in contemporary cohorts despite advances in RA management. The combination of RA and low hemoglobin resulted in a synergistic rather than additive mortality risk when compared to OA controls, suggesting that when low hemoglobin and RA coexist, they create a high-risk state exceeding their individual effects. This indicates that the presence of anemia in patients with early RA, regardless of its specific etiology, is a critical indicator of systemic vulnerability that drives adverse outcomes.

Although RA is an autoimmune disease with complex inflammatory and immunologic mechanisms, hemoglobin levels represent an integrative biomarker reflecting the net effects of systemic inflammation, nutritional status, and bone marrow function. Therefore, using baseline hemoglobin as an exposure variable to evaluate prognosis is reasonable from both biological and clinical perspectives. Unlike single inflammatory markers, hemoglobin provides a stable and readily available measure that incorporates chronic inflammatory activity and cumulative disease burden. Our findings reveal that a low hemoglobin level at RA diagnosis confers substantial mortality risk that persists across multiple analytical approaches. Sensitivity analysis suggested that despite contemporary therapeutic advances, including biological agents and treat-to-target strategies, the prognostic significance of early low hemoglobin remains unchanged. Subgroup analyses revealed important effect modifications that enhance our understanding of risk stratification. Patients without diabetes or hypertension showed stronger associations between low hemoglobin and mortality (HR: 1.75 and 1.78, respectively) compared to those with these comorbidities. This pattern suggests that in patients without competing mortality risks, hemoglobin levels provide particularly valuable prognostic information. We observed a trend toward a less pronounced association in patients with existing cardiovascular or chronic kidney disease (*p* for interaction > 0.05). This potential attenuation might reflect the already elevated baseline mortality risk in these populations, where the marginal contribution of anemia becomes proportionally smaller. The comparison with patients with OA provided additional mechanistic insights. While anemia alone increased mortality by 49% and RA alone by 40%, their combination resulted in a 130% increased risk—a clear synergistic interaction. This multiplicative effect indicates that low hemoglobin in RA represents more than the sum of its parts, likely reflecting the convergence of inflammatory pathways that both suppress erythropoiesis and accelerate atherosclerosis, endothelial dysfunction, and other systemic complications.

While the link between anemia and higher disease activity in RA is well established (11, 25, 26), often framing low hemoglobin as a surrogate for inflammatory burden, recent evidence has pivoted the focus toward mortality. For instance, a key study using the NHANES database was pivotal in confirming that anemia is associated with increased mortality risk in patients with established RA (17). Our research builds directly upon this critical finding but addresses the crucial next question: How early does this risk emerge? We uniquely demonstrated that this strong association is already present at the initial diagnosis. Furthermore, the magnitude of this risk (HR: 1.80) not only aligns with previous smaller cohorts but also provides a far more robust estimate, validated by our large-scale, matched-pair design. While we could not adjust for direct inflammatory markers such as C-reactive protein or disease activity scores, patients with newly diagnosed RA may already exhibit varying degrees of inflammation at presentation. Even without these laboratory indices, hemoglobin inherently integrates the physiological impact of inflammation, nutritional status, and bone marrow responsiveness, serving as a composite indicator of systemic stress. Given that interleukin-6–mediated hepcidin upregulation can suppress erythropoiesis (27), low hemoglobin partly reflects inflammatory burden; however, its consistent prognostic effect across subgroups suggests that hemoglobin also captures risk components not fully explained by inflammation. Thus, when inflammatory markers are unavailable, hemoglobin alone provides a practical and reliable surrogate for overall disease burden, while integrating it with C-reactive protein or erythrocyte sedimentation rate may further refine prognostic assessment in newly diagnosed rheumatoid arthritis.

Our findings have important clinical implications for early RA management. Hemoglobin measurement at diagnosis provides an immediately available and inexpensive prognostic marker that can inform treatment intensity decisions. Patients presenting with hemoglobin levels < 12 g/dl might benefit from more aggressive initial therapy, closer monitoring, and proactive cardiovascular risk management. The dose-response relationship further suggests that hemoglobin levels could be incorporated into risk stratification algorithms, with severe anemia (below 10 g/dl) potentially warranting consideration for biological therapy initiation, regardless of traditional disease activity measures.

The elevated risk of major cardiovascular events (HR: 1.47) and stroke (HR:1.25) in patients with low hemoglobin levels aligns with RA's well-established cardiovascular burden of RA. Cardiovascular disease remains the leading cause of excess mortality in RA (28, 29), and our findings suggest that baseline hemoglobin levels may identify patients at the highest risk for these complications. The pathophysiological connection likely involves multiple mechanisms: chronic inflammation drives both anemia and accelerated atherosclerosis, while reduced oxygen delivery from anemia increases the myocardial workload and may precipitate ischemic events. The stroke association, though more modest, remains clinically significant given the devastating consequences of stroke. Systemic inflammation, endothelial dysfunction, and reduced oxygen-carrying capacity from low hemoglobin levels may together increase cerebrovascular risk (30, 31). These findings support the need for comprehensive cardiovascular risk assessment and management in patients with RA presenting with low baseline hemoglobin levels, potentially including earlier statin initiation and aggressive blood pressure control.

Perhaps the most striking finding was that initial low hemoglobin strongly predicted progression to severe anemia, with nearly one-quarter of patients developing hemoglobin below 10 g/dl within 3 years (HR: 2.82). This progression pattern has critical clinical implications, as severe anemia significantly impairs quality of life and functional capacity, while increasing transfusion requirements and hospitalization risk (32, 33). Early identification of patients at risk for severe anemia progression could trigger proactive interventions, including iron supplementation, erythropoiesis-stimulating agents, and aggressive anti-inflammatory therapy to interrupt the inflammatory cascade driving worsening anemia. The dose-response relationship for the development of severe anemia (HR, 3.62; baseline hemoglobin, < 10 g/dl) suggests that even within the LHB group, further risk stratification is possible and clinically meaningful.

This study has several limitations that warrant consideration. First, the observational design precludes causal inference, although our matching strategy and sensitivity analyses strengthen the associations observed. Second, we lacked data on specific RA disease activity measures, autoantibodies, and inflammatory markers that could further clarify the mechanisms linking anemia to mortality. Third, the definition of RA relied on diagnostic codes and medication initiation rather than classification criteria, potentially introducing misclassification bias. Fourth, hemoglobin measurements were obtained within 3 months of diagnosis rather than precisely at diagnosis, possibly missing rapid early changes. Finally, our database lacked information on anemia etiology beyond iron status, preventing a detailed characterization of anemia subtypes and their differential impacts.

## 5 Conclusion

Low hemoglobin levels at RA diagnosis independently predict 3-year mortality with clear dose-response relationships, remaining prognostically significant across diverse patient subgroups and contemporary treatment eras. The synergistic interaction between RA and anemia, combined with strong associations with cardiovascular events and severe anemia progression, suggests that hemoglobin measurement provides valuable early risk stratification beyond traditional disease markers. These findings support the incorporation of hemoglobin assessment into initial RA evaluation algorithms to identify high-risk patients warranting intensified monitoring and aggressive therapeutic intervention. Future studies should investigate whether correcting early anemia through targeted interventions can modify these adverse outcomes and improve the long-term prognosis of patients with RA.

## Data Availability

The raw data supporting the conclusions of this article will be made available by the authors, without undue reservation.

## References

[1] Almutairi K Nossent J Preen D Keen H Inderjeeth C. The global prevalence of rheumatoid arthritis: a meta-analysis based on a systematic review. Rheumatol Int. (2021) 41:863–77. doi: 10.1007/s00296-020-04731-0 33175207

[2] Figus FA Piga M Azzolin I McConnell R Iagnocco A. Rheumatoid arthritis: extra-articular manifestations and comorbidities. Autoimmun Rev. (2021) 20:102776. doi: 10.1016/j.autrev.2021.102776 33609792

[3] Mitrović J Hrkač S Tečer J Golob M Ljilja Posavec A Kolar Mitrović H . Pathogenesis of extraarticular manifestations in rheumatoid arthritis-a comprehensive review. Biomedicines. (2023) 11:1262. doi: 10.3390/biomedicines11051262 37238933 PMC10216027

[4] Testa D Calvacchi S Petrelli F Giannini D Bilia S Alunno A . One year in review 2021: pathogenesis of rheumatoid arthritis. Clin Exp Rheumatol. (2021) 39:445–52. doi: 10.55563/clinexprheumatol/j1l5l3 34018918

[5] Dijkshoorn B Raadsen R Nurmohamed MT. Cardiovascular disease risk in rheumatoid arthritis anno 2022. J Clin Med. (2022) 11:2704. doi: 10.3390/jcm11102704 35628831 PMC9142998

[6] Jagpal A Navarro-Millán I. Cardiovascular co-morbidity in patients with rheumatoid arthritis: a narrative review of risk factors, cardiovascular risk assessment and treatment. BMC Rheumatol. (2018) 2:10. doi: 10.1186/s41927-018-0014-y 30886961 PMC6390616

[7] Misra DP. Clinical manifestations of rheumatoid arthritis, including comorbidities, complications, and long-term follow-up. Best Pract Res Clin Rheumatol. (2025) 39:102020. doi: 10.1016/j.berh.2024.102020 39489658

[8] Sahin D Di Matteo A Emery P. Biomarkers in the diagnosis, prognosis and management of rheumatoid arthritis: a comprehensive review. Ann Clin Biochem. (2025) 62:3–21. doi: 10.1177/00045632241285843 39242085 PMC11707974

[9] Jiang Y Zhong S He S Weng J Liu L Ye Y . Biomarkers (mRNAs and non-coding RNAs) for the diagnosis and prognosis of rheumatoid arthritis. Front Immunol. (2023) 14:1087925. doi: 10.3389/fimmu.2023.1087925 36817438 PMC9929281

[10] Abdelhafiz D Baker T Glascow DA Abdelhafiz A. Biomarkers for the diagnosis and treatment of rheumatoid arthritis - a systematic review. Postgrad Med. (2023) 135:214–23. doi: 10.1080/00325481.2022.2052626 35275765

[11] Chen YF Xu SQ Xu YC Li WJ Chen KM Cai J . Inflammatory anemia may be an indicator for predicting disease activity and structural damage in Chinese patients with rheumatoid arthritis. Clin Rheumatol. (2020) 39:1737–45. doi: 10.1007/s10067-019-04873-y 31916111

[12] Scholz GA Leichtle AB Scherer A Arndt U Fiedler M Aeberli D . The links of hepcidin and erythropoietin in the interplay of inflammation and iron deficiency in a large observational study of rheumatoid arthritis. Br J Haematol. (2019) 186:101–12. doi: 10.1111/bjh.15895 30941747

[13] Tański W Chabowski M Jankowska-Polańska B Jankowska EA. Iron metabolism in patients with rheumatoid arthritis. Eur Rev Med Pharmacol Sci. (2021) 25:4325–35. doi: 10.21203/rs.3.rs-29281/v1 34227067

[14] Song J Zhang Y Li A Peng J Zhou C Cheng X . Prevalence of anemia in patients with rheumatoid arthritis and its association with dietary inflammatory index: a population-based study from NHANES 1999 to 2018. Medicine. (2024) 103:e38471. doi: 10.1097/MD.0000000000038471 38905423 PMC11191978

[15] Chen Y Xu W Yang H Shao M Xu S Deng J . Serum levels of hepcidin in rheumatoid arthritis and its correlation with disease activity and anemia: a meta-analysis. Immunol Invest. (2021) 50:243–58. doi: 10.1080/08820139.2020.1742731 32216485

[16] Khalaf W Al-Rubaie HA Shihab S. Studying anemia of chronic disease and iron deficiency in patients with rheumatoid arthritis by iron status and circulating hepcidin. Hematol Rep. (2019) 11:7708. doi: 10.4081/hr.2019.7708 30996848 PMC6434328

[17] Dong J Yin X Zhang X Chen Z. Association between anemia and mortality in patients with rheumatoid arthritis: a retrospective cohort study of the National Health and nutrition examination survey (NHANES) database. Prev Med Rep. (2025) 54:103068. doi: 10.1016/j.pmedr.2025.103068 40336599 PMC12056958

[18] Yetişir A Sariyildiz A Türk I Coskun Benlidayi I. Evaluation of inflammatory biomarkers and the ratio of hemoglobin-red cell distribution width in patients with rheumatoid arthritis treated with tumor necrosis factor-alpha inhibitors. Clin Rheumatol. (2024) 43:1815–21. doi: 10.1007/s10067-024-06963-y 38622428

[19] Ludwig RJ Anson M Zirpel H Thaci D Olbrich H Bieber K . A comprehensive review of methodologies and application to use the real-world data and analytics platform TriNetX. Front Pharmacol. (2025) 16:1516126. doi: 10.3389/fphar.2025.1516126 40129946 PMC11931024

[20] Chen IW Yu TS Lai YC Liu PH Chang YJ Wu JY . Impact of zinc deficiency on mortality risk in patients with chronic obstructive pulmonary disease: a retrospective analysis. Front Nutr. (2025) 12:1655272. doi: 10.3389/fnut.2025.1655272 41141262 PMC12549255

[21] Hung KC Chang LC Ho CN Yu CH Lai YC Chen IW. Association between SGLT-2 inhibitors use and incidence of anemia in female patients undergoing metabolic and bariatric surgery: a retrospective study. Obes Surg. (2025) 35:4402–10. doi: 10.1007/s11695-025-08262-0 40958014

[22] Hung KC Chang LC Chang YJ Hsu CW Yew M Wu JY . Association between haemoglobin levels and the risk of diabetic retinopathy in adults with type 2 diabetes: a retrospective cohort study using the TriNetX network. Eye. (2025) 39:2822–9. doi: 10.1038/s41433-025-03982-0 40914756 PMC12494943

[23] Chen IW Chang LC Wu JY Ho CN Tsai YW Liu PH . Impact of SGLT2 inhibitors use on risk of postoperative acute kidney injury following metabolic and bariatric surgery: a retrospective study. Obes Surg. (2025) 35:3599–607. doi: 10.1007/s11695-025-08002-4 40736663

[24] Lin YL Wang SI Wei JC. Effectiveness of recombinant zoster vaccine in reducing herpes zoster incidence and all-cause mortality among patients with rheumatoid arthritis: a retrospective cohort study of 21,046 individuals from TriNetX US collaborative network. EClinicalMedicine. (2025) 85:103319. doi: 10.1016/j.eclinm.2025.103319 40656648 PMC12246860

[25] Xue L Tao L Sun H Wang Y Zhang Y Lei N . Association between blood PLT and RBC related indices and disease activity in patients with rheumatoid arthritis. Int J Gen Med. (2022) 15:573–81. doi: 10.2147/IJGM.S351505 35046715 PMC8763267

[26] Goyal L Shah PJ Yadav RN Saigal R Agarwal A Banerjee S. Anaemia in newly diagnosed patients of rheumatoid arthritis and its correlation with disease activity. J Assoc Physicians India. (2018) 66:26–9. 30477052

[27] Liu D Yan J Luo T Yang L. Association between C-reactive protein and hemoglobin in US rheumatoid arthritis patients based on NHANES data analysis. Sci Rep. (2025) 15:8905. doi: 10.1038/s41598-025-93720-z 40087374 PMC11909226

[28] Sanghavi N Ingrassia JP Korem S Ash J Pan S Wasserman A. Cardiovascular manifestations in rheumatoid arthritis. Cardiol Rev. (2024) 32:146–52. doi: 10.1097/CRD.0000000000000486 36729119

[29] Anyfanti P Ainatzoglou A Angeloudi E Michailou O Defteraiou K Bekiari E . Cardiovascular risk in rheumatoid arthritis: considerations on assessment and management. Mediterr J Rheumatol. (2024) 35:402–10. doi: 10.31138/mjr.310824.cri 39463875 PMC11500121

[30] Fonseca AC Silva DP Infante J Ferro JM. Cerebrovascular complications of anemia. Curr Neurol Neurosci Rep. (2021) 21:51. doi: 10.1007/s11910-021-01141-y 34480226

[31] Scicchitano P Cortese F Gesualdo M De Palo M Massari F Giordano P . The role of endothelial dysfunction and oxidative stress in cerebrovascular diseases. Free Radic Res. (2019) 53:579–95. doi: 10.1080/10715762.2019.1620939 31106620

[32] Finkelstein FO Finkelstein SH. The impact of anemia treatment on health-related quality of life in patients with chronic kidney disease in the contemporary era. Adv Chronic Kidney Dis. (2019) 26:250–2. doi: 10.1053/j.ackd.2019.04.003 31477255

[33] Barca-Hernando M Muñoz-Martin AJ Rios-Herranz E Garcia-Escobar I Beato C Font C . Case-control analysis of the impact of anemia on quality of life in patients with cancer: a Qca study analysis. Cancers. (2021) 13:2517. doi: 10.3390/cancers13112517 34063886 PMC8196564

