# Supplementary material for: Prognostic role of baseline hemoglobin level for long-term mortality in newly diagnosed rheumatoid arthritis: a cohort study
Source: Front Nutr. 2025 Nov 25;12:1707271. doi: 10.3389/fnut.2025.1707271 (PMC12685636; doi:10.3389/fnut.2025.1707271)
Supplement: Supplementary file 1 [file Table_1.docx]

**Table S1. ICD-10-CM Codes and Variables Used for Exposure, Matching, and Outcome Definitions**

| **Category** | **Condition / Variable** | **ICD-10-CM Code(s)** | **Notes / Description** |
| --- | --- | --- | --- |
| Exposure Definition | Rheumatoid arthritis (RA) | M05, M06 | Required diagnosis; newly diagnosed RA within TriNetX |
|  | Low hemoglobin (Hb < 12 g/dL) | — | Laboratory value within 3 months of index date |
|  | Normal hemoglobin (Hb ≥ 12 g/dL) | — | Reference cohort for comparison |
| Exclusion Criteria | Neoplasms / malignant tumors | C00–D49 | Any active malignancy |
|  | CKD stage 4 – 5 / ESRD | N18.4, N18.5, N18.6 | eGFR < 30 mL/min/1.73 m² or dialysis |
|  | Heart failure | I50 | Chronic or acute HF |
|  | Stroke / cerebral infarction | I63 | Prior ischemic stroke before index |
|  | Cirrhosis / hepatic fibrosis | K74 | Chronic liver disease |
|  | Gastrointestinal hemorrhage | K92.2 | Any upper GI bleeding |
|  | Peptic ulcer disease | K25–K28 | Gastric, duodenal, or unspecified ulcers |
| Propensity Score Matching Variables | Age (at index date) | — | Continuous variable in years |
|  | Sex (biological) | — | Male / Female |
|  | Race / ethnicity | — | Self-reported categories |
|  | Essential hypertension | I10 |  |
|  | Diabetes mellitus | E10–E14 | Includes type 1 and type 2 |
|  | Dyslipidemia / hyperlipidemia | E78 |  |
|  | Chronic kidney disease | N18 | All stages included |
|  | Coronary artery disease | I25 | Prior ischemic heart disease |
|  | Obesity / BMI ≥ 30 kg/m² | E66 |  |
|  | Chronic obstructive pulmonary disease | J44 |  |
|  | Asthma | J45 |  |
|  | Depression | F32–F33 |  |
|  | Smoking status | Z72.0 / F17 | Tobacco use history |
|  | Serum albumin | — | Nutritional / inflammatory indicator (g/dL) |
|  | Hemoglobin A1c | — | %; glycemic control |
|  | Estimated GFR | — | Renal function (mL/min/1.73 m²) |
|  | Year of index diagnosis | — | Categorical (2010–2023) |
| Outcomes | All-cause mortality | R99 / “Deceased” flag | Death recorded in TriNetX |
|  | Major adverse cardiovascular events (MACE) | I21, I46, I50 | MI, cardiac arrest, heart failure |
|  | Ischemic stroke | I63 |  |
|  | Severe anemia | — | Hb ≤ 10 g/dL during follow-up |
|  | ICU admission / critical care encounter | CPT 1013729 | Encounter code in TriNetX |
|  | Pneumonia | J12–J18 | All types of infectious pneumonia |
