# Supplementary material for: Prognostic role of baseline hemoglobin level for long-term mortality in newly diagnosed rheumatoid arthritis: a cohort study
Source: Front Nutr. 2025 Nov 25;12:1707271. doi: 10.3389/fnut.2025.1707271 (PMC12685636; doi:10.3389/fnut.2025.1707271)
Supplement: Supplementary file 2 [file Table_2.docx]

**Supplemental Table 2.** **Baseline characteristics of patients with and without low hemoglobin level before and after propensity score matching**

| Variables | Before matching | | | After matching | | | |
| --- | --- | --- | --- | --- | --- | --- | --- |
|  | LHB group  (n = 44,268) | Control group  (n = 95,078) | SMD† | | LHB group  (n = 42,267) | Control group  (n = 42,267) | SMD† |
| Antiarrhythmics | 10896 (24.6%) | 15189 (16.0%) | 0.216 | | 9736 (23.0%) | 9878 (23.4%) | 0.008 |
| Antilipemic agents | 9053 (20.5%) | 14555 (15.3%) | 0.134 | | 8232 (19.5%) | 8078 (19.1%) | 0.009 |
| Ace inhibitors | 4827 (10.9%) | 7792 (8.2%) | 0.092 | | 4388 (10.4%) | 4428 (10.5%) | 0.003 |
| Angiotensin ii inhibitor | 4393 (9.9%) | 6512 (6.8%) | 0.111 | | 3946 (9.3%) | 3886 (9.2%) | 0.005 |
| Insulins and analogues | 4052 (9.2%) | 3942 (4.1%) | 0.202 | | 3358 (7.9%) | 3124 (7.4%) | 0.021 |
| Biguanides | 2584 (5.8%) | 4386 (4.6%) | 0.055 | | 2387 (5.6%) | 2292 (5.4%) | 0.010 |
| Dipeptidyl peptidase 4 (dpp-4) inhibitors | 589 (1.3%) | 913 (1.0%) | 0.035 | | 532 (1.3%) | 522 (1.2%) | 0.002 |
| Glucagon-like peptide-1 (glp-1) analogues | 547 (1.2%) | 1254 (1.3%) | 0.007 | | 528 (1.2%) | 505 (1.2%) | 0.005 |
| Sodium-glucose co-transporter 2 (sglt2) inhibitors | 356 (0.8%) | 777 (0.8%) | 0.001 | | 338 (0.8%) | 324 (0.8%) | 0.004 |
| Antihypertensive combinations | 93 (0.2%) | 81 (0.1%) | 0.033 | | 75 (0.2%) | 64 (0.2%) | 0.006 |
